# Phasic heart rate variability and the association with cognitive performance: A cross-sectional study in a healthy population setting

**DOI:** 10.1371/journal.pone.0246968

**Published:** 2021-03-01

**Authors:** Kathrin Hilgarter, Karin Schmid-Zalaudek, Regina Csanády-Leitner, Manfred Mörtl, Andreas Rössler, Helmut Karl Lackner

**Affiliations:** 1 Division of Physiology, Otto Loewi Research Centre, Medical University of Graz, Graz, Austria; 2 Department of Obstetrics and Gynecology, Clinical Centre Klagenfurt, Klagenfurt, Austria; University of Rome Tor Vergata, ITALY

## Abstract

**Introduction:**

Sympathovagal balance measured by heart rate variability is a core component of psychophysiological research. Through the close link of physiological and psychological aspects, often a reduced heart rate variability is associated with impaired cognitive function. A better understanding of the associations between cognitive and cardiovascular dysfunctions is necessary to prevent the manifestation of diseases. Therefore, this study investigated phasic heart rate variability using rest, anticipatory, stress, and recovery periods and the association with high and low cognitive performance in a generally healthy population setting.

**Methods:**

114 healthy individuals (40 males, 74 females) aged 20 to 70 participated in the cross-sectional study. The heart rate variability based on standard deviation of NN intervals (SDNN), and the root means square of successive differences (RMSSD), low frequency (LF), high frequency (HF) and LF/HF ratio and its association with high and low cognitive performance measured by the California Verbal Learning Task II were examined.

**Results:**

The results of this study indicate that the paradigm was successful in producing stress and showed a significant association between phasic heart rate variability (SDNN) and verbal episodic memory performance, irrespective of age and sex.

**Discussion:**

The results of this study suggest that a reduced heart rate variability is associated with reduced cognitive function regardless of age and sex and seem to be an early indicator of sympathovagal disbalance.

**Conclusion:**

This leads to the conclusion that differences between high and low cognitive performance might show differences in heart rate variability at an early stage, where no diseases are yet manifest.

## 1. Introduction

Over the past few decades, psychophysiological topics have increasingly gained attention in theoretical and empirical research because the connection between physical functions and psychological processes plays an essential role in human life. From a neurophysiological point of view, several frameworks attempt to link autonomic regulation to psychological and physiological processes. The polyvagal theory [1] and the neurovisceral integration model [2] are the two main theoretical frameworks that explain the close relationship between cardiac vagal control and self-regulation. Regarding the close association between the heart and the brain, the vagus nerve received attention from researchers of both theoretical frameworks. The polyvagal theory points out the connection between autonomic regulation to psychological, physiological, and behavioral aspects from an evolutionary perspective, whereas the vagus nerve and other cranial nerves have a vital role [1, 3]. The sympathetic nervous system triggers the response to external challenges whereas the parasympathetic nervous system modulates stress recovery. This cardiac vagal tone provides an index of normal homeostatic function in non-challenging situations and adaptive functioning during challenges [3]. The neurovisceral integration model postulates a close link between prefrontal cortex and the heart through the central autonomic network and the vagus nerve. From this perspective, inhibitory processes originate within the synaptic relay mediated by the vagus nerve [2, 4, 5]. This self-regulation mechanism results in cardiac vagal control and seems to be associated with cognitive, affective, and health issues [1, 5, 6]. Both frameworks emphasize the essential role of the parasympathetic nervous system in inhibitory processes and the potential of heart rate variability (HRV) as an index of autonomic regulation of cardiac functions, which provides information whether and how an individual might handle environmental requirements [1, 2].

HRV as a physiological measure of several variables reflects the variation in the time interval (ms) between successive heartbeats and is regulated by the parasympathetic and sympathetic nervous system [7, 8], which is of interest for psychophysiological aspects. HRV is altered quickly in response to psychological stress [9] and an analysis can be performed using different methods. First, in the time domain the quantification of variability is based on the time period between successive heartbeats [10]. The most frequently used parameters are standard deviations of all NN intervals (SDNN), and square root of the mean of the sum of the squares of differences between adjacent NN intervals (RMSSD). While the RMSSD is influenced more by vagal tone, the SDNN represent activity of both sympathetic and parasympathetic branches [10–12]. Second, in the frequency domain a power spectral analysis is calculated from the stored series of data. This is an accurate method for determining the frequencies, amplitudes and other components that make up the HRV. It provides information on the magnitude of its relative intensity in the sinus rhythm of the heart [8, 11]. The power spectrum is mostly divided into low frequency (LF) 0.04–0.15 Hz and high frequency (HF) 0.15–0.4 Hz bands [7, 8]. The LF band consists of sympathetic and parasympathetic activation and might be seen as a marker of cardiac outflow [7, 12] which is influenced by baroreceptor sensitivity [12]. In contrast, the HF band is considered as an index of vagal tone [7, 12, 13]. Further, a ratio of LF/HF might be formed which reflects the activation of both the sympathetic and parasympathetic branches [8, 11, 13]. Earlier studies assumed that a LF/HF ratio provides an index of sympathovagal balance [e.g. 14]. However, in recent years this assumption faced increasing criticism because the underlying physiological principles were unclear [15], thus lowering the predictive value [12]. Third, non-linear indices like Poincaré plots, which consist of a visual presentation of time series signals, can be used to analyze changes in HRV. The standard deviation of the Poincaré plot perpendicular to the line-of-identity (SD1) represents the short-term variability, the standard deviation along the line-of-identity represents the long-term variability (SD2) [16]. However, the non-linear metrics SD1 and SD2 are described in the existing literature as identical to the RMSSD and SDNN [17].

Stress as an event considered threatening by an individual triggers a physiological and behavioral stress response [18]. If the human body is stressed long-term or in frequent intervals, the stress system can malfunction [19] and an altered autonomic regulation can be the result, which may contribute to the worsening of a person’s cardiovascular [4, 20] and cognitive health [21]. Executive cognitive functions originating in the dorsolateral prefrontal cortex, including working memory processes, attention, cognitive control processes, and mental flexibility, among others, represent crucial aspects to handle environmental information and perform everyday life tasks [22, 23]. From this perspective, executive cognitive functions might be a sensitive marker for early autonomic [24] or cardiovascular [20] dysfunctions.

The exposure to short-term mental stress reduces parasympathetic nervous system activity through a reduction of acetylcholine and allows for increases in basal sympathetic activity by increases in epinephrine and norepinephrine in healthy individuals [25–27]. During mental stress, mean HR increases [26], mean RR-interval and RMSSD [25] decrease, and additionally, LF/HF and LF tend to increase [25, 26]. Respiratory sinus arrhythmia does not change under short-term stress [26]. The findings of Standard Deviation of R-R interval (SDRR) are inconsistent. Both increased [26] and decreased [25] SDRR during acute mental stress were reported.

Previous evidence suggests that a reduced HRV precedes the development of several risk factors [24] and is often associated with cardiovascular risk events, mortality, or other morbidities [28–32]. Further associated factors include age, sex, lifestyle (smoking, lower physical activity, overweight), chronic stress, and psychological disorders [33–38]. Most of these risk factors are also associated with impaired cognitive functioning [39–42]. This suggests that reduced HRV and cognitive impairment share some common risk factors. However, age and sex are the main factors which influence HRV. For instance, both HRV [43] and cognition [41, 44] decrease with age. A linear decline in SDNN and a U-shaped pattern in RMSSD have been reported [45]. Moreover, females have higher mean HR, less variability in SDNN and reduced power in LF, but higher power in HF compared to males [46] and show differential associations between HRV and psychological variables [47]. Moreover, a recent systematic review reported a close association between the autonomous nervous system and the neurocognitive system [48]. This close association might play an essential role in both physical and mental health. In this context, age-related physiological or psychological disorders seem to have an enormous public health impact [49].

Within the experimental paradigm in previous studies, two different concepts were analyzed, the tonic and phasic HRV. Tonic HRV is observed at a one-time point and is commonly indicated by resting HRV. In contrast, phasic HRV investigates the system’s reactivity at different time points frequently indexed by resting, stress, and recovery [12]. With respect to this differentiation, most studies investigated tonic HRV [50–57] and showed that individuals with higher tonic HRV tended to cope better with stress [58] and showed better cognitive or executive performance [53, 56, 57], and vice versa, a link between lower tonic HRV levels and lower performance [50, 52, 54, 55] was reported. Nevertheless, another study observed that resting HRV was not associated with cognitive performance [51]. However, a close link between higher tonic and enhanced phasic HRV was reported [59]. Only a few studies observed the association of phasic HRV and cognitive performance [60–63]. However, there were inconsistent HRV changes, and most of them did not consider age or sex as recommended [38]. Overall, there is missing evidence about how the physiological system reacts indicated by HRV in the context of cognitive performance irrespective of age and sex. A better understanding of reaction patterns between cognitive and cardiovascular dysfunctions at an early stage might be necessary to take preventive measures before diseases manifest. Therefore, the aim of this study is to investigate how HR and HRV, indicated by SDNN, RMSSD, LF, HF, LF/HF react with rest, anticipation, stress, and recovery, considering high and low verbal episodic memory performance in a generally healthy population setting, irrespective of age and sex.

## 2. Materials and methods

### 2.1. Participants

Overall, 134 participants were recruited for the study. Consistent with prior recommendations [10, 12], the following inclusion criteria were applied to select healthy participants: no existing cardiovascular disease with or without a history of hypertension, no chronic disease with a cardiovascular component such as hypertension, no self-reported psychological disorder (e.g. major depressive disorder), no history of addiction such as drug or alcohol dependence, no cardiovascular prescription medication usage, no competitive athletes, no existing pregnancy in women, and a body mass index (BMI) of less than 35 kg/m^2^. The health status was self-reported. In total, 19 participants were excluded: 14 were under medication or had an acute infection, 3 showed a pathological electrocardiogram, 1 participant (19 years old) did not meet the criteria for age which required a minimum age of 20 years, and 1 exceeded the BMI >35 kg/m^2^. Based on artifact control, 1 participant was further excluded from the analysis. The final cohort comprised 114 participants (40 males, 74 females; all Caucasian) aged 20 to 70 years (mean = 36.8±12.5). Participants were requested to abstain from alcohol for 24 hours and from caffeine and nicotine for a minimum of four hours prior to their testing. The study was in accordance with the 1964 Declaration of Helsinki and was approved by the authorized local ethics committee (EK-28-511 ex 15/16; EK-A23/16). Informed written consent was obtained from all participants prior to their participation in the study. Demographic characteristics of the final cohort are detailed in **Table 1**. The strict inclusion criteria were based on the most recently updated recommendations in the literature for HRV [10, 12].

**Table 1:** Demographic characteristics of the participants.

Variable	Male		Female		Total	
	n = 40		n = 74		n = 114	
Age in years (mean ± SD)	38.7±13.6		35.81±11.8		36.8±12.5	
BMI in kg/m^2^ (mean ± SD)	24.59±3.18		22.69±2.81		23.36±3.07	
Cigarette smokers	**n**	**(%)**	**n**	**(%)**	**n**	**(%)**
No	27	(23.68)	46	(40.35)	73	(64.03)
Yes	9	(7.90)	17	(14.91)	26	(22.81)
n/a	4	(3.51)	11	(9.65)	15	(13.16)
Education						
Secondary education first stage	5	(4.39)	4	(3.51)	9	(7.90)
Secondary education second stage	13	(11.40)	39	(34.21)	52	(45.61)
Tertiary education	18	(15.79)	20	(17.54)	38	(33.33)
n/a	4	(3.51)	11	(9.65)	15	(13.16)
Age frequency distribution						
20–29 years	18	(15.79)	40	(35.09)	58	(50.88)
30–39 years	6	(5.26)	8	(7.02)	14	(12.28)
40–49 years	5	(4.39)	16	(14.04)	21	(18.42)
50–59 years	7	(6.14)	7	(6.14)	14	(12.28)
≥60 years	4	(3.51)	3	(2.63)	7	(6.14)

### 2.2. Procedure

**Fig 1:**
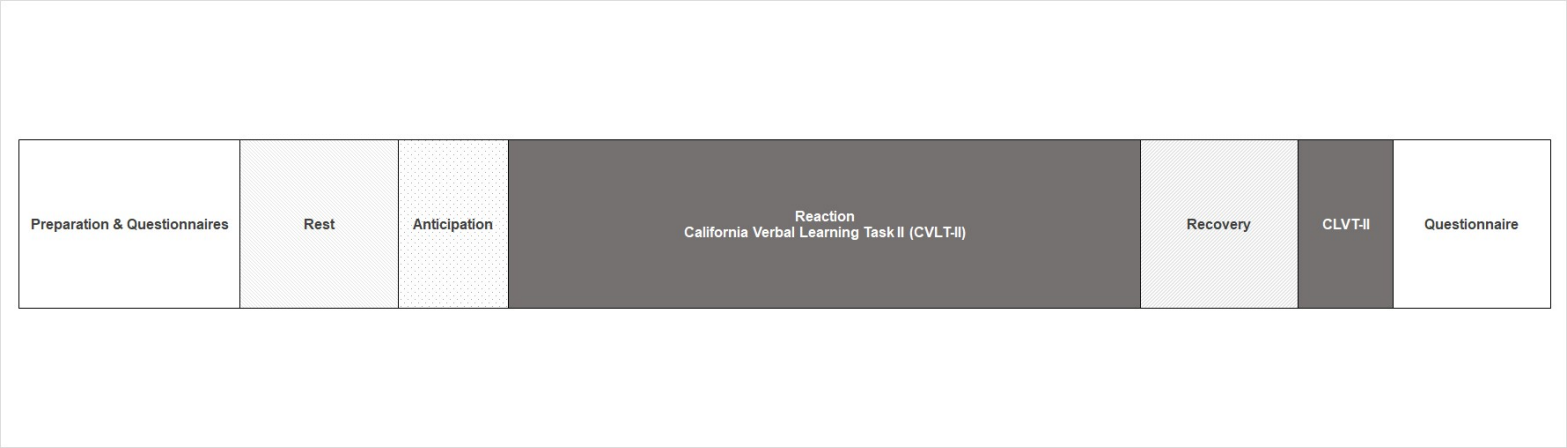
Experimental procedure.

After the assessment of demographic (age, sex, the highest level of education) and clinical data (self-reported health status, weight, height, waist and hip circumference), the electrodes were placed. Participants were seated in a comfortable chair in a silent room. The detailed procedure has been described before [64]. A 5-min baseline period (1^st^ phase–rest) was followed by a 3-min anticipation period (2^nd^ phase–anticipation). Thereafter, a cognitive stressor period (3^rd^ phase–stress) was executed. The study design consists of both an emotionally stressful element (anticipation phase) and a stressful cognitive element (verbal learning task). After the induced stress, a 5-min recovery period (4^th^ phase–recovery) was performed. An overview of the experimental procedure is shown in **Fig 1**.

#### 2.2.1. Emotional and cognitive stressor

Emotional stressor (anticipation): Participants were instructed that the ageing process can affect the functioning of the brain. Since the memory function is a sensitive indicator of such ageing processes, mental memory is presented, and the results of this test will be evaluated by colleagues in the psychiatric department who determine whether memory performance matches their age or already indicates early brain ageing. After this instruction, the 3-min anticipation period started.

Cognitive stressor: A standardized verbal memory task–the California Verbal Learning Task II (CVLT-II) [65], German adaptation [66]–was used as a mild stressor and also to assess the cognitive episodic verbal learning performance of the participants. Individuals were required to memorize as many words as possible from a list of 16 words read to them for 20-seconds via headphones. Thereafter, a 30 s preparation period followed, during which a numerical counter on the computer screen counted from 30 to 1, indicating when the participant was to start reproducing the words. Participants were given a minimum of 30 s to recall and recite as many words as they remembered. This process was repeated five times and represents the immediate recall–list A (Trial 1–5). After the 5^th^ run, the same procedure was repeated with another list of words (list B: interference list), representing the immediate free recall. The request to recite word list A was repeated after a 30-second waiting time and without the list being read out to participants in advance. This is referred to as the short delay free recall (SDFR). After a break of 20-minutes, in which no verbal test was performed, the request to recite for word list A was again repeated without the list being read out in advance. Participants were once more encouraged to respond verbally after 30 s, which is referred to as the long delay free recall (LDFR). For analysis of the cognitive episodic verbal learning performance, the CVLT variables Trial 1 (T1), total recall (TR) representing the sum of Trial 1 to Trial 5, SDFR, and LDFR were used. The mean processing time of the CVLT-II consisted of 30:89±3:92 minutes.

#### 2.2.2. Additional data collection

Physical activity was assessed using the Freiburg Questionnaire of physical activity–short form. This self-assessment tool for recording activity behavior provides information about basic, leisure, and training activity in terms of time and energy consumption [67]. In addition, participants completed the general depression scale [68] and some other questionnaires on the computer, which are not relevant to the present research question. Furthermore, participants retrospectively rated their subjective perceived difficulty on a 17-point rating scale ranging from ‘not difficult at all’ to ‘extremely difficult’ after the cognitive stress task.

### 2.3. Recording of HRV

Continuous hemodynamic monitoring of HR (3-lead electrocardiography, sampling rate = 1000 Hz) as well as oscillometric and continuous blood pressure (sampling rate = 100 Hz) were carried out with the Task Force® Monitor (TFM®; CNSystems, Graz, Austria; Certification CE [TÜV-A-MT- 1/10/1Q034]) [69]. Data processing was fully automated by MATLAB (MathWorks Natick, Massachusetts, MA, United States) [70, 71]. For the purpose of this study, the cardiovascular data of four periods (rest, anticipation, stress, and recovery) were analyzed. HR [bpm] and HRV were indicated by using the frequency domain with *ln*(LF) [ms^2^], *ln*(HF) [ms^2^], LH/HF ratio and the time domain with SDNN [ms], RMSSD [ms].

### 2.4. Statistical analysis

Overall, descriptive statistics are presented as mean ± standard deviation. The differences in cognitive, psychological, and HRV parameters at the baseline were analyzed within groups such as sex (male, female), education (secondary education first stage, secondary education second stage, tertiary education), perceived task difficulty (the median split in difficulty and no difficulty), smoking (yes, no), self-reported overall fitness (the median split in higher and lower fitness level), and age. One-way analysis of variance for physiological variables and Kruskal-Wallis test or Mann-Whitney-U test for cognitive (CVLT-II) variables were performed. The effects of cognitive performance on HRV were examined by separate repeated-measures ANCOVAs using the time (rest, anticipation, stress, recovery) as within-subject factors; sex and different cognitive high- and low-performance groups as between-subject factors, and age as a covariate. The high and low groups of cognitive performance were created by a median split of CVLT-II variables (T1, SDFR, LDFR, TR). Specifically, subjects were classified into groups with each CVLT-II variable investigated in this study. High and low values represent better and worse cognitive performance, respectively. All variables except LF, HF, and LF/HF ratio were within the range of a normal distribution. Hence, these parameters were log-transformed for further analysis. Analogous to those described above, supplementary analyses (ANCOVAs) were conducted with delta’s in HR and HRV, with adjusting for baseline values. Finally, supplementary analyses were done to explore correlations between HR and HRV variables and demographic characteristics such as BMI, fitness, and smoking as well as cognitive performance variables such as T1, SDRF, LDFR, and TR using Pearson’s correlation coefficient. The Greenhouse-Geisser adjustment was used to correct for violations of sphericity. For pairwise comparisons, the Bonferroni correction was applied to test the statistical significance. All significant effects were evaluated with partial eta squared (η^2^) as an indicator of effect size. A two-tailed significance level of α = 0.05 was used for all analyses. All analyses were conducted using IBM SPSS Statistics for Windows (Version 26, Armonk, NY: IBM Corp.).

## 3. Results

### 3.1. General cognitive and psychological parameters

Analysis of the retention of information over short and long intervals showed that females (F) memorized significantly more words compared with males (M) in T1 (Median_F_ = 7.0; Median_M_ = 7.0), TR (Median_F_ = 60.0; Median_M_ = 52.5), SDFR (Median_F_ = 13.0; Median_M_ = 12.0), and LDFR (Median_F_ = 14.0; Median_M_ = 12.0), exact Mann-Whitney-U test: U_T1_ = 1107.50, *p* = 0.024, U_TR_ = 885.50, *p* < 0.001, U_SDFR_ = 1095.00, *p* = 0.021, and U_LDFR_ = 1045.00, *p* = 0.009. Those participants who found the task more difficult (D), defined by median split, showed lower memory performance in SDFR (Median_E_ = 14.0; Median_D_ = 13.0), LDFR (Median_E_ = 14.0; Median_D_ = 12.0) and TR (Median_E_ = 62.0; Median_D_ = 55.0) compared to participants who found the task easier (E), exact Mann-Whitney-U test: U_SDFR_ = 1256.00, *p* = 0.036, and U_TR_ = 1137.00, *p* = 0.006, U_LDFR_ = 1196.00, *p* = 0.015. No significant effects were found between cognitive performance variables, smoking status, and self-reported overall fitness. Furthermore, a significant age-related difference in cognitive performance was observed in TR [H(4) = 14.55, *p* = 0.006], SDFR [H(4) = 21.37, *p* < 0.001], and LDFR [H(4) = 22.70, *p* < 0.001]. Subsequent post hoc tests indicated that the youngest age group “20–29 years” differed significantly from the groups “≥60 years” (*z*_*SDFR*_ = 13.09, *p* = 0.003, *r* = 1.23; *z*_*LDFR*_ = 13.09, *p* = 0.010, *r* = 1.23; *z*_*TR*_ = 13.21, *p* = 0.017, *r* = 1.24), “50–59 years” (*z*_*SDFR*_ = 9.75, *p* = 0.003, *r* = 0.91; *z*_*LDFR*_ = 9.74, *p* = 0.003, *r* = 0.91; *z*_*TR*_ = 8.41, *p* = 0.006, *r* = 0.79), and “40–49 years” (*z*_*SDFR*_ = 8.33, *p* < 0.001, *r* = 0.78; *z*_*LDFR*_ = 8.33, *p* < 0.001, *r* = 0.78; *z*_*TR*_ = 9.83, *p* = 0.014, *r* = 0.92). No significant effects were found in cognitive performance variables and the limited classification of education (secondary education first stage, secondary education second stage, tertiary education). The scores for psychological parameters showed a normal range (M = 7.86 ± 6.09) as per the Center for Epidemiologic Studies Depression Scale (CES-D).

### 3.2. General HRV parameters

Females showed significantly higher values in *ln*(HF) and RMSSD than males at the baseline, whereas the LF/HF ratio was higher in males compared with females. All HRV variables were associated with age except for baseline HR. However, participants who were smokers had a significantly higher HR at baseline than non-smokers. No significant effects were found between HR or HRV variables and education, task difficulty, or self-reported overall fitness at baseline, as shown in **Table 2**. Furthermore, no noteworthy correlations between delta HR/HRV variables and demographic characteristics such as BMI, fitness, and smoking were found (see S1 Table). The results of several correlation analyses between delta HR/HRV variables and cognitive performance parameters are shown in S2 Table.

**Table 2:** Study sample characteristics.

Variables	Absolute values	*Cognitive performance*	
**Cognitive performance parameters**		***high performance***	***low performance***
Trial 1 (T1)	7.08±1.60^a^^k^	*8*.*71±0*.*73*	*6*.*01±0*.*99*
Total recall (TR)	56.82±9.29^a^^,^^b^^,^^d^	*63*.*34±4*.*31*	*48*.*18±6*.*69*
Short delay free recall (SDFR)	12.67±2.86^a^^,^^b^	*14*.*61±1*.*21*	*10*.*00±2*.*24*
Long delay free recall (LDFR)	12.82±2.86^a^^,^^b^^,^^d^	*14*.*79±1*.*76*	*10*.*02±2*.*10*
**Psychological parameters**			
CES-D	7.86±6.09		
**Physiological parameter at baseline**		***Cognitive performance in T1***	
***Frequency domain***		***high performance***	***low performance***
*ln*(LF) [ms^2^]	6.31±1.05^b^	*6*.*47±0*.*96*	*6*.*21±1*.*11*
*ln*(HF) [ms^2^]	5.80±1.31^a^^,^^b^	*6*.*12±1*.*18*	*5*.*60±1*.*36*
LF/HF ratio [–]	0.51±1.06^a^^,^^b^	*0*.*36±1*.*18*	*0*.*61±0*.*97*
***Time domain***			
HR [bpm]	70.89±10.31^e^	*70*.*48±10*.*32*	*71*.*16±10*.*37*
SDNN [ms]	45.92±20.87^b^	*49*.*93±19*.*95*	*43*.*30±21*.*18*
RMSSD [ms]	36.39±21.98^a^^,^^b^	*41*.*05±23*.*05*	*33*.*35±20*.*86*

Absolute values are mean ± standard deviation, n = 114, *p* < 0.05.

^a^ Main effect of sex

^b^ Main effect of age

^c^ Main effect of education

^d^ Main effect of task difficulty

^e^ Main effect of smoking

^f^ Main effect of self-reported overall fitness.

Since blood pressure (BP) is a major autonomic and cognitive confounder, differences in resting BP were observed between high and low performers. However, no significant effects were found in systolic blood pressure [*F*_(1,113)_ = 1.25, *p* = 0.265], diastolic blood pressure [*F*_(1,113)_ = 2.29, *p* = 0.746], and mean arterial blood pressure [*F*_(1,113)_ = 1.55, *p* = 0.216].

### 3.3. Stress-induced HRV and the association to cognitive performance

**Fig 2:**
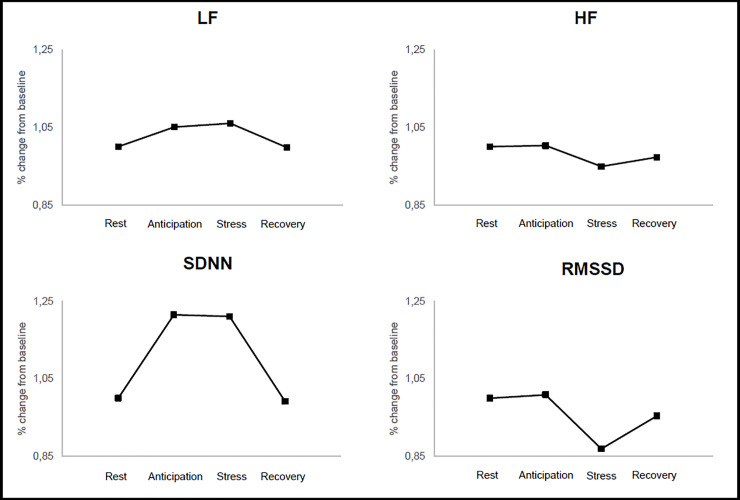
HRV changes during period of rest, anticipation, stress, and recovery.

The repeated-measures ANCOVA indicated statistically significant differences of mean HRV parameters between periods (rest, anticipation, stress, recovery), irrespective of age and sex; RMSSD [*F*_(2.29,254.15)_ = 11.00, *p* < 0.001, η^2^ = 0.09], *ln*(HF) [*F*_(2.51,278.84)_ = 8.96, *p* < 0.001, η^2^ = 0.08], LF/HF [*F*_(2.55,283.22)_ = 6.09, *p* < 0.001, η^2^ = 0.05], HR [*F*_(1.66,184.56)_ = 22.33, *p* < 0.001, η^2^ = 0.17], and SDNN [*F*_(2.76,306.25)_ = 7.66, *p* < 0.001, η^2^ = 0.07]. Bonferroni-corrected pairwise comparisons revealed that SDNN and *ln*(LF) significantly increased from the baseline to the anticipatory phase (*p* < 0.001) and significantly decreased from the stress task phase to the recovery phase (*p* < 0.001). No significant difference was observed between the anticipatory phase and the stress task phase or between the baseline and the recovery phase (*p* > 0.999). *Ln*(HF) and RMSSD decreased significantly from the anticipatory phase to the stress task phase (*p* < 0.001), while *ln*(HF) differed significantly from the baseline to the recovery phase (*p* = 0.010). No significant difference between the baseline and the anticipatory phase (*p* > 0.999) or between the stress task phase and the recovery phase was observed (*p* > 0.320). HR and LF/HF increased significantly from the baseline to the anticipatory phase (*p* < 0.001), increased significantly from the anticipatory phase to stress task phase (*p* < 0.001), and decreased significantly from the stress task phase to the recovery phase (*p* < 0.001). No significant difference between baseline and recovery period was noted (*p* = 0.078). **Fig 2** provides an overview of the changes during all periods of relevant HRV variables. An age-related interaction effect of SDNN [*F*_(2.76,306.25)_ = 3.13, *p* = 0.030, *η^2^* = 0.03], RMSSD [*F*_(2.29,254.15)_ = 6.67, *p* < 0.001, *η^2^* = 0.06], and HF[*F*_(2.51,278.84)_ = 4.26, *p* = 0.009, *η^2^* = 0.04] as well as a sex-related interaction effect in *ln*(HF) [*F*_(2.51,278.84)_ = 2.86, *p* = 0.047, *η^2^* = 0.03] was observed. From the significant interaction in *ln*(HF) between period and sex, additional pairwise comparison shows that the reaction pattern between males and females differs significantly. While there is only a significant difference between anticipation and recovery in males (*p* = 0.029), in females there is a clear difference between baseline and stress, anticipation and stress as well as between baseline and anticipation and recovery. In addition, females have significantly higher scores in all periods (baseline: *p* < 0.001; anticipation: *p* < 0.001; stress: *p* = 0.021; recovery: *p* < 0.001).

**Fig 3:**
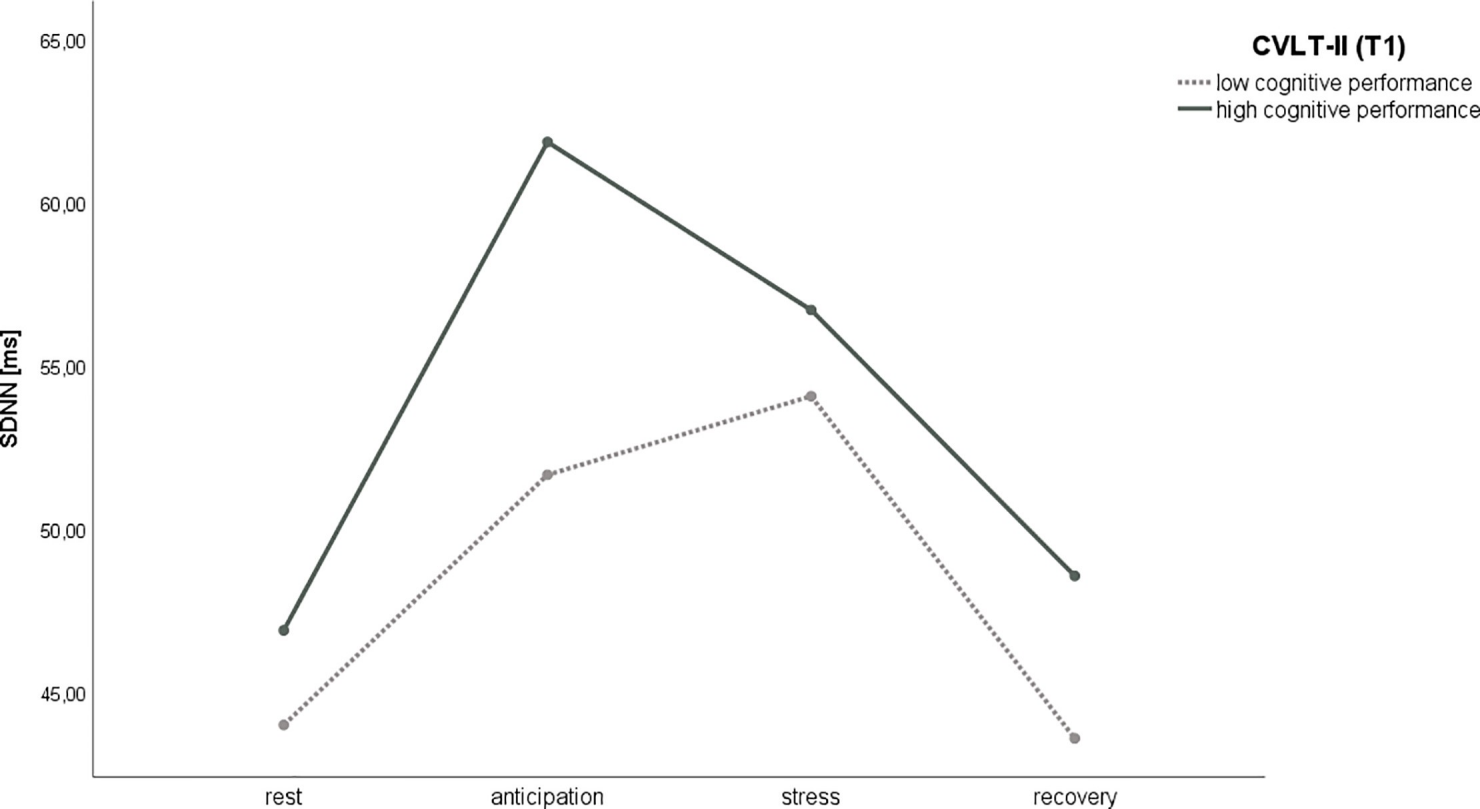
SDNN relative to high and low cognitive performance.

A significant interaction by high and low cognitive performance (T1) irrespective of age and sex driven by higher SDNN [*F*_(2.74,298.26)_ = 3.01, *p* = 0.035, *η^2^* = 0.03] during the anticipation phase was found, as shown in **Fig 3**. The results of the transition from rest to anticipation (*p* < 0.001) and stress to recovery (*p* < 0.001) are significant in both performance groups, while the results of the transition from anticipation to stress are not significant in the low performance (*p* = 0.946) or high performance group (*p* = 0.314). The results of the transition from rest to recovery are not significant in both performance groups (*p* = 1.000), which means that both come back close to baseline levels. Furthermore, a trend toward a significant interaction by high and low cognitive performance (T1) irrespective of age and sex driven by higher HR [*F*_(1.68,182.90)_ = 3.10, *p* = 0.056, *η^2^* = 0.03] during the cognitive stressor period was found. No other significant difference between time points in *ln*(LF) [*F*_(2.36,262.01)_ = 1.10, *p* = 0.349] or between high or low levels of cognitive performance in HRV were found. **Table 3** provides an overview of all effects. In addition, similar results for adjusted baseline by using delta values are shown in S3 Table.

**Table 3:** Overview of all effects.

Origin	Variable	Rest	Anticipation	Stress	Recovery	F statistics	
**Frequency domain**	*ln*(LF) [ms^2^]	6.31±1.05	6.63±0.93	6.69±0.77	6.30±0.92	period	*F*_(2.36,262.01)_ = 1.10, *p* = 0.349, η^2^ = 0.01
						period x age	*F*_(2.36,262.01)_ = 1.65, *p* = 0.188, η^2^ = 0.02
						period x sex	*F*_(2.36,262.01)_ = 0.14, *p* = 0.903, η^2^ = 0.00
	*ln*(HF) [ms^2^]	5.80±1.31	5.82±1.26	5.51±1.09	5.65±1.30	period	***F***_**(2.51,278.84)**_ **= 8.96, *p* < 0.001, η^2^ = 0.08**
						period x age	***F***_**(2.51,278.84)**_ **= 4.26, *p* = 0.009, η^2^ = 0.04**
						period x sex	***F***_**(2.51,278.84)**_ **= 2.86, *p* = 0.047, η^2^ = 0.03**
	LF/HF [–]	0.51±1.06	0.81±0.94	1.19±0.78	0.66±1.01	period	***F***_**(2.55,283.22)**_ **= 6.09, *p* < 0.001, η^2^ = 0.05**
						period x age	*F*_(2.55,283.22)_ = 1.38, *p* = 0.252, η^2^ = 0.01
						period x sex	*F*_(2.55,283.22)_ = 1.61, *p* = 0.195, η^2^ = 0.01
**Time domain**	SDNN [ms]	45.92±20.87	55.78±23.15	55.59±19.75	45.51±19.18	period	***F***_**(2.76,306.25)**_ **= 7.66, *p* < 0.001, η^2^ = 0.07**
						period x age	***F***_**(2.76,306.25)**_ **= 3.13, *p* = 0.030, η^2^ = 0.03**
						period x sex	*F*_(2.76,306.25)_ = 0.53, *p* = 0.650, η^2^ = 0.01
						period x T1	***F***_**(2.74,298.26)**_ **= 3.01, *p* = 0.035, η^2^ = 0.03**
	RMSSD [ms]	36.39±21.98	36.70±21.70	31.64±17.46	34.73±22.07	period	***F***_**(2.29,254.15)**_ **= 11.00, *p* < 0.001, η^2^ = 0.09**
						period x age	***F***_**(2.29,254.15)**_ **= 6.67, *p* < 0.001, η^2^ = 0.06**
						period x sex	*F*_(2.29,254.15)_ = 2.75, *p* = 0.058, η^2^ = 0.02
-	HR [bpm]	70.89±10.31	72.73±10.69	79.06±12.07	71.67±10.39	period	***F***_**(1.66,184.56)**_ **= 22.33, *p* < 0.001, η^2^ = 0.17**
						period x age	*F*_(1.66,184.56)_ = 2.13, *p* = 0.130, η^2^ = 0.02
						period x sex	*F*_(1.66,184.56)_ = 2.93, *p* = 0.066, η^2^ = 0.03
					Trend	period x T1	*F*_(1.68,182.90)_ = 3.10, *p* = 0.056, η^2^ = 0.03

## 4. Discussion

In the current study, an investigation of phasic HRV during periods of rest, anticipation, stress, and recovery in relation to high and low verbal episodic memory performance in a generally age- and sex-matched healthy population setting was carried out.

The results indicate that the applied stress paradigm produced stress and was appropriate to examine different physiological changes due to psychological challenges. The HR and HRV indices indicated by SDNN, RMSSD, *ln*(HF), and LF/HF changed significantly during different periods and were a consequence of short-term psychological stress. This suggests that HRV indices are sensitive to any psychological changes in different periods of the stress response, irrespective of age and sex. The data also confirm previous studies describing the alteration of HRV during psychological stress [9, 72–74].

The findings of the present study provide insights into physiological reactivity patterns to stress-inducing tasks. As reported in this study, an increased cardiac activity indexed by increased HR, SDNN, and LF/HF and a vagal withdrawal as indicated by a decreased RMSSD and *ln*(HF) during stress compared to the baseline were observed. These physiological patterns in response to psychological stress are congruent with previous research where evidence suggested a reduction of HF combined with increasing LF/HF and HR in professors before and after a lecture [75]. Similar results were reported during the Stroop test [72] and during the verbal answering of questions [76]. Moreover, some authors reported a decreasing RMSSD during working memory tasks or chess problems [61, 62]. Furthermore, an increasing SDNN during the Trier Social Stress Test and speaking tasks [26, 63] or a decreasing SDNN during stressful chess problems compared to baseline [62] were reported. The results of this study support the evidence which indicates that RMSSD and HF is influenced more by vagal tone, whereas HR and SDNN represent activity of both branches [7, 10–13]. However, vagal withdrawal is the most frequently reported effect in the context of phasic HRV and psychological stress [25, 26, 74].

From a neurophysiological view, the successful adaptation of social behavior depends on the withdrawal and re-establishment of the vagal brake which represents a mechanism to support metabolic requirements which change in response to environmental challenges [1, 3]. Accordingly, vagal withdrawal is lower in sustained attention to maintain social behavior than in fight or flight responses, which can be accompanied by a complete vagal withdrawal [1]. A decreasing phasic HRV indicated by RMSSD and *ln*(HF) during cognitive stress, such as in this study, indicates the withdrawal of cardiac vagal control and triggers a defensive system to handle the cognitive stressor [77]. From a structural perspective, the association between psychological factors, such as emotion and cognition, and physiological processes might be explained by a common reciprocal inhibitory cortico-subcortical neural circuit [2]. Moreover, previous studies show that changes in HRV are accompanied by changes in the cerebral blood flow in those areas that are important for self-regulation, including executive functioning, stress management, emotion regulation, social functioning, and inhibitory processes [5, 78]. As shown experimentally, HRV is linked to self-regulation effort [59]. Regardless, self-regulation supports the organism in mobilizing physiological resources and in enabling goal-directed behavior in response to changing demands [2]. Regarding the demands of a situation, both an increasing or decreasing HRV reactivity seem to be adaptive [1, 6]. As reported in this study, the pattern of sympathetic activation combined with vagal withdrawal during psychological stress might be explained by the mobilization of self-regulatory abilities. Regarding this perspective, self-regulation of cardiac vagal withdrawal and re-establishment is linked to cognitive load [59] and indicates that the reactivity depends on the task characteristics and situation [79]. Evidence suggests that the utilization of self-regulation resources is indicated by a larger vagal withdrawal, which represents the adaptive function [5, 59] when individuals are facing a physical or mental stressor [12] to provide and mobilize energy for the organism to handle the stressor [1].

As reported in this study, LF/HF ratio increased during cognitive stress. This indicates sympathetic activity and parasympathetic inhibition, which occurs when facing a stressor and is connected to fight-or-flight behavior. However, the interpretation of LF/HF ratio as an index of sympathovagal balance must be interpreted with caution because of the complicated nature of LF power, the weak relationship to the activation of the sympathetic nervous system, and the non-linear and often non-reciprocal relationship between sympathetic and parasympathetic nerve activity which is influenced by effects of respiration and HR [15].

The different reaction patterns of both sexes in HF, which is parasympathetically driven, suggest that in this sample, females are more vagally regulated by vagal withdrawal during cognitive stress. Moreover, females show higher values in all periods. This is in line with existing meta-analysis where females show higher values in HF [46]. Regarding these results, the effects of hormones like estrogen [80] and genetic factors like brain structures [81] have been discussed to explain the differences in HRV between males and females.

In contrast to the other HRV (SDNN, RMSSD, *ln*(HF), LF/HF) indices in this study, the *ln*(LF) was strongly influenced by age and sex, although it did not differ significantly between the main time points. This might suggest a higher sensitivity of *ln*(LF) to influencing factors during different periods.

Overall, the current study observed the differences between higher and lower cognitive performance groups during episodic verbal learning along with different periods of HRV irrespective of age and sex. Accordingly, both better and worse performance groups showed an increased SDNN in the anticipatory period (emotional stressor). However, this effect was stronger in the higher-performing group than the lower-performing group, even though the baseline values were approximately the same in both groups. These findings show that higher phasic HRV (SDNN) during acute psychological short-time stress was significantly associated with a better cognitive performance in an episodic verbal learning task and thus support the idea that cognitive functions, including working memory, are closely linked to physiological processes. As showed experimentally, the larger the cardiac vagal withdrawal from rest to task, the fewer math errors were made [79]. This might be explained by the close relationship between the autonomic nervous system and certain centers in the brain that are also responsible for cognitive performance [6, 60]. Evidence suggests that resting HRV might predict phasic HRV [59]. Therefore, the results of this study are supported by several experimental studies which reported that lower resting HRV indicates worse autonomic function [53, 62] and worse cognitive performance [82]. Furthermore, studies observed associations between resting SDNN, LF, and LF/HF and lower values in the Montreal Cognitive Assessment [52] or between reduced RMSSD, NN50, and HF and cognitive impairment as ascertained by the Mini-Mental State Examination [50]. In both studies, results were irrespective of confounders. In a clinical setting, a lower resting SDNN is an established risk factor for mortality among post-myocardial infarction patients [83], type 2 diabetes mellitus [31], and might also be a predictor of mortality [30].

The different reaction patterns of high and low cognitive performance groups in the anticipatory period might also be based on different appraisals of the task as postulated by the neurovisceral integration model [2]. Based on the results of the current study, it seems that a reduced phasic SDNN in the context of emotional stress (anticipatory period) is associated with lower cognitive performance, irrespective of age and sex, whereas higher values of SDNN during acute emotional stress are associated with better cognitive performance. Previous studies observed a close association between successful anticipatory emotion regulation and increased phasic HRV. Authors reported that trait rumination levels during stress moderate the effect of anticipatory emotion regulation (ER) strategies during HRV [84]. Therefore, humans show a better coping with stress when they use reappraisal within the anticipatory phase [84]. The neurocognitive framework for regulation expectation postulates that a high ability to regulate stressful situations is based on a high actual self-esteem combined with a low ideal self-esteem. In addition, this is related to high expectation and high tendency to accept unsuccessful coping and is associated with increased proactive control. Accordingly, this leads to predictive dorsolateral prefrontal cortex activity and decreased amygdala activity [85]. The results of this study suggest that the different reaction patterns indicated by higher values of SDNN in the anticipation period within the higher performing group might reflect a different approach to the regulation of anticipatory stress and emotions compared to low performers. Evidence suggests that during stressful situations that require the regulation of emotions, which depends on top-down functions, phasic HRV increases [5]. Further, the results of this study indicate that this anticipatory process of the high performers serves as an adaptive function in dealing with a memory performance task to reduce the impact of a potential threat. Previous studies have shown that effective anticipatory stress regulation requires proactive cognitive control in combination with the activity of the prefrontal cortex [85]. Further, the proactive anticipation of induced emotional stress is also associated with lower cognitive effort during stress [86]. Consequently, it is assumed that higher HRV is linked to higher activation of the prefrontal cortex and better performance, such as enhanced working memory and sustained attention [6]. However, more research is needed to determine the exact psychophysiological mechanisms behind successful stress anticipation in connection with cognitive performance.

In accordance with the present results, higher SDNN was cross‐sectionally associated with better performance of working memory [87]. Regarding cognitive performance, a study reported that HRV, especially SDNN, pNN50, and RMSSD were significantly higher in high-performance individuals during medium and high-level chess problems relative to their low-performance counterparts [62]. There are several possible explanations of how HRV might influence cognitive functions. The interaction of the sympathetic and parasympathetic nervous system enables the body to adapt to changing requirements in order to satisfy the different needs of the altered blood supply. Some authors have speculated that a sympathovagal imbalance results in a higher blood pressure variability and might be associated with pathological changes in the cardiovascular system [53]. The pathological changes, such as brain lesions caused by hypertension, might underlie the cognitive impairment [88].

During recovery, both the higher- and lower-performing group return to approximately SDNN baseline levels. These results indicate that during a stress condition, the decrease or increase in SDNN might be interpreted as a reaction of stress, corresponding to the well-known phenomenon that a healthy heart will adapt to changing demands. A higher HRV indexed by SDNN in response to emotional stress exists more in the higher cognitive performance group than in the lower cognitive performance group. The SDNN provides the total HRV and might be used as an index of physiological stress resilience. Therefore, the higher and irregular the HRV, the higher the SDNN and the better the stress resilience [74].

Overall, the results of the present study indicate that a reduced HRV indexed by SDNN is associated with reduced cognitive function irrespective of age or sex and might be seen as an early indicator of sympathovagal disbalance. Further studies, particularly longitudinal studies, are necessary to observe and better understand the ongoing changes and processes from both the physiological and the neuropsychological perspective. Moreover, task-induced decreases in HF band, such as in this study, were linked to changes in regional cerebral blood flow to cortical and subcortical brain regions which seems to regulate cardiac autonomic activity with cognitive and emotional behavioral processes [89]. Given that these differences in HRV during emotional and cognitive tasks exist, more investigation from a neurophysiological perspective is needed.

Finally, a few important limitations of the study need to be considered. Related to the use of self-reported information to screen for a relatively healthy population, it cannot be guaranteed that none of the subjects had any underlying diseases. Further, the generalizability of this study is limited due to the healthy population setting. Moreover, the heterogenic and small sample size of this study must also be considered; therefore, transferability might be limited. Furthermore, the results must be interpreted with caution because HRV indices like SDNN depend on the length of the recordings; thus, comparability with other studies is restricted. Finally, females and younger adults are predominant in this study compared to males or older adults. However, this might also be seen as a strength, since most studies investigate older adults in the context of HRV and cognitive performance.

## 5. Conclusion

In this cross-sectional study of healthy asymptomatic participants across all ages and both sexes, a significant association between phasic HRV and verbal episodic memory performance was observed. Predominantly, a higher phasic HRV (SDNN) is associated with higher cognitive performance, irrespective of age and sex. HRV indices like SDNN seem to be a sensitive marker to any change in the mental state. This indicates that high and low cognitive performance groups might also show a difference in HRV at an early stage, where no diseases are yet manifest. Individuals with higher proactive anticipation of induced emotional stress, indicated by higher HRV (SDNN), show a better cognitive performance. The adaptation of the cardiovascular system to psychological stress is based on a close interaction between the sympathetic and parasympathetic branches of the nervous system and HRV is an indicator of physiological adaptation to current requirements. This enables a differentiated mapping of the processes involved in stress reactivity and suggests a close interaction between HRV, anticipatory ER, and cognitive performance.

## Supporting information

S1 Table Pearson’s correlation coefficients between delta HR/HRV variables and demographic characteristics. (DOCX)

S2 Table Pearson’s correlation coefficients between delta HR/HRV and cognitive performance variables. (DOCX)

S3 Table Overview of all effects based on delta values. (DOCX)
