# Supplementary material for: Phasic heart rate variability and the association with cognitive performance: A cross-sectional study in a healthy population setting
Source: PLoS One. 2021 Mar 1;16(3):e0246968. doi: 10.1371/journal.pone.0246968 (PMC7920382; doi:10.1371/journal.pone.0246968)
Supplement: S1 Table — (DOCX) [file pone.0246968.s001.docx]

**S1 Table. Pearson’s correlation coefficients between delta HR/HRV variables and demographic characteristics.**

| **Variables** | ***1*** | ***2*** | ***3*** | ***4*** | ***5*** | ***6*** | ***7*** | ***8*** | ***9*** | ***10*** | ***11*** | ***12*** | ***13*** | ***14*** | ***15*** | ***16*** | ***17*** | ***18*** | ***19*** | ***20*** | ***21*** |
| --- | --- | --- | --- | --- | --- | --- | --- | --- | --- | --- | --- | --- | --- | --- | --- | --- | --- | --- | --- | --- | --- |
| **ΔHR_A (1)** | 1 | ,616** | ,496** | 0,184 | -0,183 | -,219* | -,505** | -,441** | -,409** | -0,015 | -,249** | -0,142 | -,260** | -,260** | -0,155 | 0,161 | -0,046 | -0,016 | -0,001 | 0,016 | -0,033 |
| **ΔHR_S (2)** |  | 1 | ,469** | 0,105 | -0,147 | -0,124 | -,342** | -,564** | -,330** | -0,045 | -,278** | -0,054 | -0,111 | -,352** | -0,156 | 0,035 | -0,005 | 0,068 | -0,116 | 0,007 | -0,042 |
| **ΔHR_R (3)** |  |  | 1 | 0,127 | -,200* | -,301** | -,283** | -,247** | -,523** | -0,066 | -,261** | -,220* | -,263** | -,262** | -,434** | 0,117 | -0,057 | 0,125 | 0,109 | ,255** | -0,085 |
| **ΔSDN_A (4)** |  |  |  | 1 | ,414** | ,408** | ,256** | -0,019 | -0,012 | ,491** | ,355** | ,289** | ,272** | 0,040 | 0,109 | ,247** | ,316** | ,191* | 0,043 | -0,026 | -0,083 |
| **ΔSDN_S (5)** |  |  |  |  | 1 | ,593** | ,361** | ,459** | ,266** | ,310** | ,719** | ,434** | ,318** | ,480** | ,239* | 0,059 | ,338** | ,229* | 0,002 | 0,032 | 0,144 |
| **ΔSDN_R (6)** |  |  |  |  |  | 1 | ,348** | ,294** | ,549** | ,303** | ,481** | ,690** | ,286** | ,292** | ,479** | 0,074 | ,248** | ,287** | 0,039 | -0,020 | 0,027 |
| **ΔRMS_A (7)** |  |  |  |  |  |  | 1 | ,645** | ,478** | ,262** | ,294** | ,244** | ,633** | ,436** | ,320** | -,195* | -0,043 | -0,015 | 0,071 | 0,039 | -0,054 |
| **ΔRMS_S (8)** |  |  |  |  |  |  |  | 1 | ,431** | 0,041 | ,363** | 0,143 | ,292** | ,703** | ,218* | -0,159 | -0,178 | -0,031 | 0,076 | -0,011 | 0,070 |
| **ΔRMS_R (9)** |  |  |  |  |  |  |  |  | 1 | 0,034 | 0,169 | ,305** | ,318** | ,363** | ,696** | -0,183 | -0,109 | -,247** | 0,086 | -0,124 | -0,015 |
| **ΔLF_A (10)** |  |  |  |  |  |  |  |  |  | 1 | ,555** | ,505** | 0,180 | 0,104 | 0,117 | ,754** | ,463** | ,392** | 0,106 | -,191* | 0,016 |
| **ΔLF_S (11)** |  |  |  |  |  |  |  |  |  |  | 1 | ,592** | ,227* | ,357** | 0,147 | ,333** | ,706** | ,450** | 0,020 | -0,038 | 0,144 |
| **ΔLF_R (12)** |  |  |  |  |  |  |  |  |  |  |  | 1 | ,247** | 0,169 | ,343** | ,276** | ,450** | ,688** | 0,083 | -0,003 | 0,015 |
| **ΔHF_A (13)** |  |  |  |  |  |  |  |  |  |  |  |  | 1 | ,449** | ,490** | -,511** | -0,118 | -0,143 | 0,101 | -0,005 | 0,030 |
| **ΔHF_S (14)** |  |  |  |  |  |  |  |  |  |  |  |  |  | 1 | ,429** | -,209* | -,409** | -0,170 | 0,128 | -0,049 | 0,034 |
| **ΔHF_R (15)** |  |  |  |  |  |  |  |  |  |  |  |  |  |  | 1 | -,226* | -0,181 | -,445** | 0,115 | -0,126 | -0,005 |
| **ΔLFHF_A (16)** |  |  |  |  |  |  |  |  |  |  |  |  |  |  |  | 1 | ,484** | ,438** | 0,026 | -0,163 | -0,006 |
| **ΔLFHF_S (17)** |  |  |  |  |  |  |  |  |  |  |  |  |  |  |  |  | 1 | ,569** | -0,078 | 0,000 | 0,119 |
| **ΔLFHF_R (18)** |  |  |  |  |  |  |  |  |  |  |  |  |  |  |  |  |  | 1 | -0,01 | 0,096 | 0,019 |
| **BMI (19)** |  |  |  |  |  |  |  |  |  |  |  |  |  |  |  |  |  |  | 1 | -0,027 | 0,157 |
| **Fitness (20)** |  |  |  |  |  |  |  |  |  |  |  |  |  |  |  |  |  |  |  | 1 | -0,124 |
| **Smoking (21)** |  |  |  |  |  |  |  |  |  |  |  |  |  |  |  |  |  |  |  |  | 1 |

ΔA is based on differences between anticipation period and baseline; ΔS is based on differences between stress period and baseline; ΔR is based on differences between recovery period and baseline.

** The correlation is significant at the level of 0.01 (2-sided).

* The correlation is significant at the level of 0.05 (2-sided).
