# Supplementary material for: Phasic heart rate variability and the association with cognitive performance: A cross-sectional study in a healthy population setting
Source: PLoS One. 2021 Mar 1;16(3):e0246968. doi: 10.1371/journal.pone.0246968 (PMC7920382; doi:10.1371/journal.pone.0246968)
Supplement: S2 Table — (DOCX) [file pone.0246968.s002.docx]

**S2 Table. Pearson’s correlation coefficients between delta HR/HRV and cognitive performance variables.**

| **Variables** | ***1*** | ***2*** | ***3*** | ***4*** | ***5*** | ***6*** | ***7*** | ***8*** | ***9*** | ***10*** | ***11*** | ***12*** | ***13*** | ***14*** | ***15*** | ***16*** | ***17*** | ***18*** | ***19*** | ***20*** | ***21*** | ***22*** |
| --- | --- | --- | --- | --- | --- | --- | --- | --- | --- | --- | --- | --- | --- | --- | --- | --- | --- | --- | --- | --- | --- | --- |
| **ΔHR_A (1)** | 1 | ,616** | ,496** | 0,184 | -0,183 | -,219* | -,505** | -,441** | -,409** | -0,015 | -,249** | -0,142 | -,260** | -,260** | -0,155 | 0,161 | -0,046 | -0,016 | 0,168 | ,192* | 0,050 | 0,121 |
| **ΔHR_S (2)** |  | 1 | ,469** | 0,105 | -0,147 | -0,124 | -,342** | -,564** | -,330** | -0,045 | -,278** | -0,054 | -0,111 | -,352** | -0,156 | 0,035 | -0,005 | 0,068 | ,249** | ,235* | ,189* | ,211* |
| **ΔHR_R (3)** |  |  | 1 | 0,127 | -,200* | -,301** | -,283** | -,247** | -,523** | -0,066 | -,261** | -,220* | -,263** | -,262** | -,434** | 0,117 | -0,057 | 0,125 | 0,137 | 0,142 | 0,071 | 0,122 |
| **ΔSDN_A (4)** |  |  |  | 1 | ,414** | ,408** | ,256** | -0,019 | -0,012 | ,491** | ,355** | ,289** | ,272** | 0,040 | 0,109 | ,247** | ,316** | ,191* | ,209* | ,205* | ,235* | ,206* |
| **ΔSDN_S (5)** |  |  |  |  | 1 | ,593** | ,361** | ,459** | ,266** | ,310** | ,719** | ,434** | ,318** | ,480** | ,239* | 0,059 | ,338** | ,229* | -0,050 | -0,02 | 0,066 | -0,053 |
| **ΔSDN_R (6)** |  |  |  |  |  | 1 | ,348** | ,294** | ,549** | ,303** | ,481** | ,690** | ,286** | ,292** | ,479** | 0,074 | ,248** | ,287** | -0,023 | -0,012 | 0,010 | -0,087 |
| **ΔRMS_A (7)** |  |  |  |  |  |  | 1 | ,645** | ,478** | ,262** | ,294** | ,244** | ,633** | ,436** | ,320** | -,195* | -0,043 | -0,015 | 0,036 | 0,018 | 0,066 | 0,032 |
| **ΔRMS_S (8)** |  |  |  |  |  |  |  | 1 | ,431** | 0,041 | ,363** | 0,143 | ,292** | ,703** | ,218* | -0,159 | -0,178 | -0,031 | -0,090 | -0,142 | -0,08 | -0,146 |
| **ΔRMS_R (9)** |  |  |  |  |  |  |  |  | 1 | 0,034 | 0,169 | ,305** | ,318** | ,363** | ,696** | -0,183 | -0,109 | -,247** | 0,031 | -0,086 | -0,113 | -0,035 |
| **ΔLF_A (10)** |  |  |  |  |  |  |  |  |  | 1 | ,555** | ,505** | 0,180 | 0,104 | 0,117 | ,754** | ,463** | ,392** | 0,105 | 0,032 | 0,085 | 0,113 |
| **ΔLF_S (11)** |  |  |  |  |  |  |  |  |  |  | 1 | ,592** | ,227* | ,357** | 0,147 | ,333** | ,706** | ,450** | -0,159 | -0,101 | 0,007 | -0,121 |
| **ΔLF_R (12)** |  |  |  |  |  |  |  |  |  |  |  | 1 | ,247** | 0,169 | ,343** | ,276** | ,450** | ,688** | -0,077 | -0,059 | 0,028 | -0,057 |
| **ΔHF_A (13)** |  |  |  |  |  |  |  |  |  |  |  |  | 1 | ,449** | ,490** | -,511** | -0,118 | -0,143 | 0,021 | -0,06 | 0,033 | 0,012 |
| **ΔHF_S (14)** |  |  |  |  |  |  |  |  |  |  |  |  |  | 1 | ,429** | -,209* | -,409** | -0,17 | -0,081 | -,230* | -0,180 | -,206* |
| **ΔHF_R (15)** |  |  |  |  |  |  |  |  |  |  |  |  |  |  | 1 | -,226* | -0,181 | -,445** | 0,111 | -,187* | -,202* | -0,062 |
| **ΔLFHF_A (16)** |  |  |  |  |  |  |  |  |  |  |  |  |  |  |  | 1 | ,484** | ,438** | 0,078 | 0,068 | 0,053 | 0,090 |
| **ΔLF HF_S (17)** |  |  |  |  |  |  |  |  |  |  |  |  |  |  |  |  | 1 | ,569** | -0,094 | 0,076 | 0,143 | 0,038 |
| **ΔLFHF_R (18)** |  |  |  |  |  |  |  |  |  |  |  |  |  |  |  |  |  | 1 | -0,159 | 0,088 | 0,182 | -0,006 |
| **T1 (19)** |  |  |  |  |  |  |  |  |  |  |  |  |  |  |  |  |  |  | 1 | ,289** | ,275** | ,484** |
| **SDFR (20)** |  |  |  |  |  |  |  |  |  |  |  |  |  |  |  |  |  |  |  | 1 | ,693** | ,623** |
| **LDFR (21)** |  |  |  |  |  |  |  |  |  |  |  |  |  |  |  |  |  |  |  |  | 1 | ,533** |
| **TR (22)** |  |  |  |  |  |  |  |  |  |  |  |  |  |  |  |  |  |  |  |  |  | 1 |

ΔA is based on differences between anticipation period and baseline; ΔS is based on differences between stress period and baseline; ΔR is based on differences between recovery period and baseline.

** The correlation is significant at the level of 0.01 (2-sided).

* The correlation is significant at the level of 0.05 (2-sided).
