# Supplementary material for: Phasic heart rate variability and the association with cognitive performance: A cross-sectional study in a healthy population setting
Source: PLoS One. 2021 Mar 1;16(3):e0246968. doi: 10.1371/journal.pone.0246968 (PMC7920382; doi:10.1371/journal.pone.0246968)
Supplement: S3 Table — (DOCX) [file pone.0246968.s003.docx]

**S3 Table. Overview of all effects based on delta values.**

| **Origin** | **Variable** | **ΔA** | **ΔS** | **ΔR** | **F statistics** | |
| --- | --- | --- | --- | --- | --- | --- |
| **Frequency domain** | *ln*(LF) [ms²] | 0.32±0.54 | 0.38±0.89 | -0.01±0.64 | period | *F*_(1.88,205.37)_ = 0.81, *p* = 0.447, η² = 0.01 |
|  |  |  |  |  | period x age | *F*_(1.88,205.37)_ = 2.47, *p* = 0.090, η² = 0.02 |
|  |  |  |  |  | period x sex | *F*_(1.88,205.37)_ = 0.06, *p* = 0.930, η² = 0.00 |
|  | *ln*(HF) [ms²] | 0.02±0.41 | -0.29±0.69 | -0.16±0.51 | period | ***F*_(1.81,197.73)_ = 8.25, *p* < 0.001, η² = 0.07** |
|  |  |  |  |  | period x age | ***F*_(1.81,197.73)_ = 3.18, *p* = 0.049, η² = 0.03** |
|  |  |  |  |  | period x sex | *F*_(1.81,197.73)_ = 1.78, *p* = 0.174, η² = 0.02 |
|  | LF/HF [-] |  |  |  | period | *F*_(2,218)_ = 2.35, *p* = 0.098, η² = 0.02 |
|  |  |  |  |  | period x age | *F*_(2,218)_ = 0.76, *p* = 0.468, η² = 0.01 |
|  |  |  |  |  | period x sex | *F*_(2,218)_ = 1.41, *p* = 0.246, η² = 0.01 |
| **Time domain** | SDNN [ms] | 9.86±12.34 | 9.67±14.39 | -0.41±10.96 | period | ***F*_(2,218)_ = 10.25 *p* < 0.001, η² = 0.09** |
|  |  |  |  |  | period x age | ***F*_(2,218)_ = 3.18, *p* = 0.044, η² = 0.03** |
|  |  |  |  |  | period x sex | *F*_(2,218)_ = 0.21, *p* = 0.814, η² = 0.00 |
|  |  |  |  |  | period x T1 | ***F*_(2,218)_ = 3.61, *p* = 0.029, η² = 0.03** |
|  | RMSSD [ms] | 0.31±6.85 | -4.75±13.42 | -1.66±9.66 | period | ***F*_(1.72,187.51)_ = 11.07, *p* < 0.001, η² = 0.09** |
|  |  |  |  |  | period x age | ***F*_(1.72,187.51)_ = 5.40, *p* = 0.008, η² = 0.05** |
|  |  |  |  |  | period x sex | *F*_(1.72,187.51)_ = 1.73, *p* = 0.184, η² = 0.02 |
| ~~-~~ | HR [bpm] | 1.83±3.14 | 8.17±7.04 | 0.78±3.23 | period | ***F*_(1.44,157.34)_ = 23.75, *p* < 0.001, η² = 0.18** |
|  |  |  |  |  | period x age | *F*_(1.44,157.34)_ = 1.55, *p* = 0.219, η² = 0.01 |
|  |  |  |  |  | period x sex | *F*_(1.44,157.34)_ = 1.27, *p* = 0.276, η² = 0.01 |
|  |  |  |  | Trend | period x T1 | *F*_(1.44,157.34)_ = 3.25*, p* = 0.057, *η²* = 0.03 |

ΔA is based on differences between anticipation period and baseline; ΔS is based on differences between stress period and baseline, ΔR is based on differences between recovery period and baseline.
